# Involvement of Polo-like kinase 1 (Plk1) in quiescence regulation of cancer stem-like cells of the gastric cancer cell lines

**DOI:** 10.18632/oncotarget.16839

**Published:** 2017-04-05

**Authors:** Lin Zhu, Sheng Xing, Li Zhang, Jian-Min Yu, Cheng Lin, Wei-Jun Yang

**Affiliations:** ^1^ College of Life Sciences, Zhejiang University, Hangzhou 310058, People's Republic of China

**Keywords:** PLK1, RSK1, cancer stem cell, quiescence, drug and radiation resistance

## Abstract

Cancer stem cells (CSCs) have been hypothesized to initiate tumor growth and be resistant to chemoradiotherapy, and these processes appear to be closely related to CSC quiescence. Here, a CSC-like cell population with a high level of CD44 expression was obtained from the human gastric cancer cell lines MKN45 and MKN74. Using a PKH26-labeling retention assay, quiescent CSC-like cells with low levels of Ki67 and PCNA expression were found in spheres formed in serum-free medium, and exhibited resistance to drug and radiation treatments. Polo-like kinase 1 (Plk1) and ribosomal S6 kinase 1 (RSK1) were silenced in the quiescent CSC-like cells. The Plk1-specific inhibitors inhibited the activation of RSK1 and induced quiescence in the CSC-like cells, but increased RSK1 activity and resulted in apoptosis in non-CSCs. Furthermore, RSK1 silencing by inhibitors activated Plk1 and had no effect on the growth of spheres in the CSC-like cells, but did not affect phosphorylation of Plk1 and led to decreased proliferation in non-CSCs. Our results showed that Plk1 and RSK1 play important roles in the conversion of CSCs between active and quiescent states.

## INTRODUCTION

Cancer stem cells (CSCs) are defined as “cells within a tumor that possess the capacity for self-renewal and that can cause the heterogeneous lineages of cancer cells that constitute the tumor” [1]. They were first demonstrated in acute myeloid leukemia by Dick and coworkers in 1997, and were subsequently identified in a variety of cancers, including brain, breast, and gastric cancers [2–5]. Based on the expression of cell surface markers, such as CD44 and CD133, CSCs have been isolated from gastric cancer cell lines, lung tumor tissues, and other cancers [5–8]. Increasing evidence reveals that CSCs are responsible not only for tumor initiation, invasion, and metastasis, but also for relapse. Furthermore, several characteristics make them highly resistant to traditional cancer therapies, including relatively slow cycling, powerful DNA repair function, high expression of drug transporters, and apoptosis resistance [9–10]. CSCs from oral squamous cell carcinoma (OSCC) show increased migration, invasion, and malignancy both *in vitro* and *in vivo*, and expression of Nanog/Oct-4/CD133 is higher in OSCC patients with the worst survival prognosis [11]. Spheroid colony formation is often used to identify CSCs in human brain tumors, melanomas, and glioblastomas [3, 12, 13], and is also an efficient method to identify CSCs in gastric cancer [5, 14]. Furthermore, CSCs can be enriched in spheres in renal cell carcinoma and OSCC [15, 16]. CSCs provides us with a framework for further understanding carcinogenesis and for explaining the high failure rate of treatments and the poor survival rate of gastric cancer patients.

Cell quiescence, which is a reversible non-proliferative state with decreased transcription and translation levels and low metastasis, is an important property of CSCs [17–19]. Evidence of CSC quiescence has been found in breast cancer and acute myeloid leukemia [20–22]. Similar to stem cells, which are able to exit the quiescent state to differentiate and proliferate in response to stress, quiescent leukemic stem cells enter the cell cycle after chemotherapy [17]. More and more evidence suggests that quiescence in CSCs is a crucial mechanism for their chemoradiotherapy resistance and survival, which in turn can eventually lead to cancer relapse [23, 24]. Using a long-term PKH26-labeling retention assay, quiescent stem cells were identified among human normal mammary stem cells and in hepatocellular carcinoma [21, 25], epithelial ovarian cancer, and other cancers [26, 27]. However, knowledge of the mechanism underlying CSC quiescence is limited. Therefore, greater understanding of this mechanism is crucial for developing novel CSC-targeted therapies to decrease treatment failure rates and cancer relapse.

Polo-like kinase 1 (Plk1) is a serine/threonine protein kinase belonging to the human Polo-like kinase family [28]. Due to the importance of Plk1 in cell proliferation, its expression and activation are tightly regulated during the cell cycle [29, 30]. However, Plk1 is highly overexpressed in non-small cell lung cancer, human head and neck cancer, and in patients with a poor prognosis, suggesting that overexpression of Plk1 promotes carcinogenesis and is closely associated with cancer aggression [31, 32]. Knockdown or depletion of Plk1 by RNA interference inhibits tumor growth in nude mouse models [33]. Therefore, Plk1 has drawn great attention and been regarded as a potential anti-cancer target in cancer therapeutic research. As an upstream regulator of Plk1, Aurora A (AURKA, also known as STK6) activates Plk1 through phosphorylation of a conserved threonine residue (Thr-210), and activation of Plk1 is required for mitotic entry and progression [34, 35]. Plk1 inhibits the mitogen-activated protein kinase kinase-extracellular signal-regulated kinase-ribosomal S6 kinase 1 (MEK-ERK-RSK1) cascade in *Artemia* embryonic mitosis and HeLa cells [36]. RSK1 promotes cell cycle progression and cell proliferation in different stages of the cell cycle by phosphorylating a variety of substrates implicated in cell division [37, 38].

Recently, specific inhibitors of protein kinases in Plk1 and RSK1 signaling pathways have been used to study cell cycle regulation, including some which are expected to be clinically used as anti-cancer agents. BI 2536 specifically inhibits Plk1 functions in mitosis, which suppresses cell division and increases apoptosis in HeLa cancer cells [39]. Inhibition of RSK1 using the specific inhibitor SL0101 suppresses the proliferation of human prostate cancer and breast cancer cells [40, 41]. However, no data have been published concerning how CSCs are regulated by Plk1 and RSK1 and their related inhibitors and associated signaling pathways.

In the present study, we identified and isolated CSC-like cells from the gastric cancer cell lines MKN45 and MKN74 by fluorescence-activated cell sorting (FACS) analysis of CD44 and an *in vitro* sphere formation assay, and targeted quiescent CSC-like cells in spheres. We found that Plk1 and other kinases involved in the MEK-ERK-RSK1 signaling cascade were all inactivated in the quiescent CSC-like cells. Inhibition of Plk1 using specific inhibitors suppressed the division of CSC-like cells, and led to inactivation of the MEK-ERK-RSK1 pathway. In addition, activation of Plk1 was significantly increased in RSK1-inhibited CSC-like cells, suggesting that RSK1 inhibits Plk1 in the CSC-like cells. However, the situation was different in the cancer cells: inhibition of Plk1 activated RSK1 via its upstream kinase MEK/ERK, and Plk1 activity was not affected by RSK1 suppression.

## RESULTS

### Isolation and characterization of CSC-like cells from the human gastric cancer cell lines

**Figure 1:**
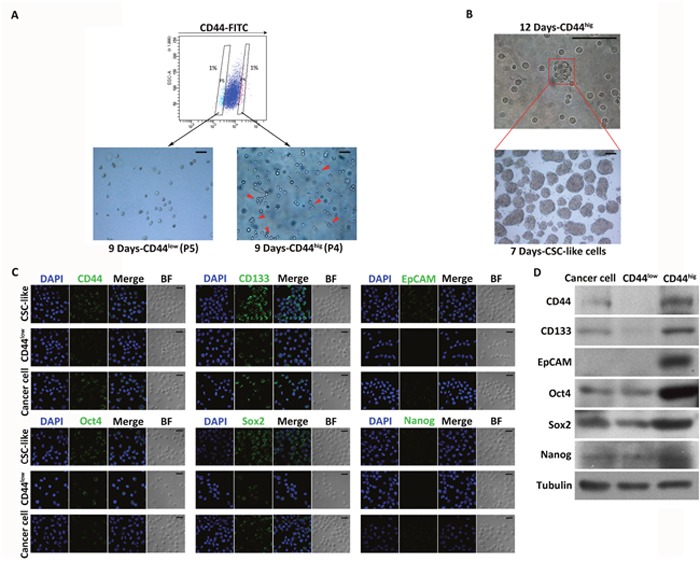
Isolation and characterization of CSC-like cells from MKN45 cells **(A)** FACS analysis of CD44 on MKN45 cells. Percentages in the flow plot indicate the cells with the lowest (P5) and highest (P4) expression of CD44 isolated from the analyzed populations (top). Images show sorted P5 (CD44^low^) and P4 (CD44^hig^) cells cultured in serum-free medium for 9 days (bottom). Growing spheres were only found among CD44^hig^ cells (indicated by red arrows). Scale bars, 50 μm. **(B)** An example of a spheroid colony (indicated by red arrows) formed by a single CD44^hig^ cell cultured for 12 days (top). Images show spheres originating from single cells dissociated from spheroid colonies formed by CD44^hig^ cells (bottom). Scale bars, 100 μm. **(C)** Expression profiles of stem cell markers (CD44, CD133, EpCAM, Oct4, Sox2, and Nanog) in CD44^hig^, CD44^low^, and unsorted cells detected by immunofluorescence. DAPI stained the cell nuclei (blue). BF = bright field. Scale bars, 20 μm. **(D)** Expression profiles of stem cell markers in CD44^hig^, CD44^low^, and unsorted cells detected by Western blot.

To isolate CSC-like cells, we analyzed the expression pattern of CD44 by performing FACS of the cell line MKN45. Based on the expression level of CD44, two main populations were distinguished. The CD44^hig^ subpopulation accounted for 1% of the analyzed cells and had the highest expression level of CD44. The CD44^low^ subpopulation accounted for 1% of the analyzed cells and had the lowest expression level of CD44 (Figure 1A).

The CD44^hig^ and CD44^low^ subpopulations sorted from MKN45 cells were cultured under non-adherent conditions in serum-free medium containing EGF and bFGF. After 9 days of incubation, cells in the CD44^hig^ population proliferated and generated small (immature) spherical colonies, whereas there was no detectable sphere formation by CD44^low^ cells (Figure 1A). The spheres formed by CD44^hig^ cells were maintained in culture for 12 days and then disaggregated, and the single cells obtained were cultured. After 7 days of incubation, about 64.85±4.32% (n=3) of the cells produced spheres, indicating a strong self-renewal ability (Figure 1B). During the culture period, only spheres were collected for passaging, and 54.34±5.83% (n=20) of cells disaggregated from spheres were able to form spheres in every passage, which were maintained for 20 passages.

CD44 high (CD44^hig^) and low expression (CD44^low^) cells were also sorted from MKN74 cells and then cultured in sphere-forming conditions for 10 days. 60.01±9.33% (n=5) of the CD44^hig^ MKN74 cells formed spheres, while no spheres were found in the CD44^low^ MKN74 cells (Supplementary Figure 1A). To further confirm the CSC properties of the spheroid cells, western blot and immunofluorescence analysis were performed to evaluate the protein expression levels of CD44, CD133, epithelial cell adhesion molecule (EpCAM), Oct4, Sox2, and Nanog, which are key molecular markers to identify CSCs and play major roles in stemness maintenance, transcription, and nuclear reprogramming [5, 11, 14, 42–44]. Expression not only of CD44 but of all the other markers, especially CD133, Oct4 and Sox2, was much higher in spheroid cells than in CD44^low^ and unsorted cells (Figure 1C and 1D, Supplementary Figure 1B).

### Quiescent cells in spheres formed by CSC-like cells and their resistance to treatments with chemical drugs and radiation

**Figure 2:**
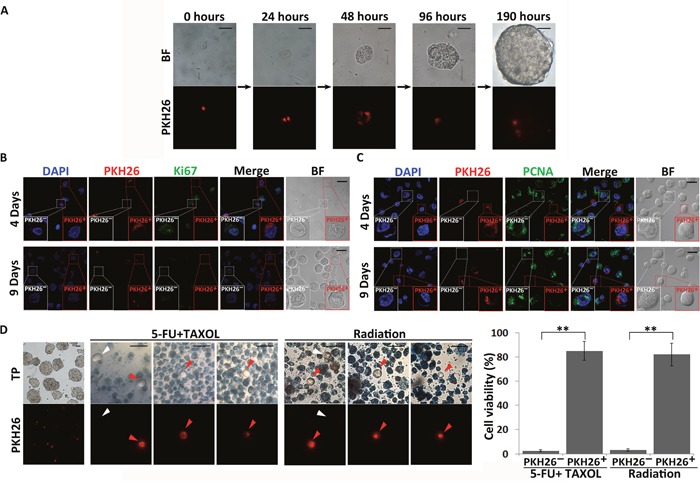
Identification of quiescent CSC-like cells in spheres **(A)** The PKH26-labeling retention assay was performed upon MKN45 CSC-like cell sphere formation. Single CSC-like cells were labeled with PKH26 and cultured in serum-free medium for 8 days. Images were taken at the indicated time points. The red signals indicate PKH26 staining. BF = bright field. Scale bars, 25 μm. **(B)** and **(C)** Expression of proliferation markers (Ki67 and PCNA) in PKH26^+^ and PKH26^-^ CSC-like cells. DAPI stained the cell nuclei (blue). Red signals indicate PKH26 staining. Green signals indicate expression of Ki67 and PCNA. BF = bright field. Scale bars, 20 μm. Experiments were repeated four times. **(D)** Quiescent CSCs are resistant to drug and radiation treatments. Leftmost column shows spheres originating from single PKH26-labeled cells before drug (1 mM 5-FU and 10 μM TAXOL) and direct radiation (30 Gy) treatments. Red signals indicate PKH26 staining. Cells were stained with trypan blue after treatment. Red and white arrows indicate PKH26^+^ and PKH26^-^ cells not stained with trypan blue, respectively. TP = trypan blue. Scale bars, 30 μm. The number of viable cells was quantitatively analyzed from three independent experiments. All data represent the mean ± S.E. **, P < 0.05.

To identify quiescent CSC-like cells in the spheres, we performed a PKH26-labeling retention assay upon CSC-like cell sphere formation. PKH26-labeled CSC-like cells were plated, and the process of sphere formation and concomitant PKH26-staining statuses were recorded for 8 days. With continuous cell division and growth, the intensity of red staining on the cell membrane was gradually dispersed and reduced. After 4 days of culture, a few cells in spheres remained brightly labeled with PKH26 stain, which displayed a much higher staining intensity than in the other cells (Figure 2A and Supplementary Figure 1C). Moreover, with the growth of spheres, PKH26 staining was sustained for 8 days. Therefore, we proposed that these PKH26-retaining (PKH26^+^) CSC-like cells are in a non-dividing and quiescent state.

To further confirm the quiescent state of PKH26^+^ CSC-like cells, the expression of the proliferation markers Ki67 and proliferating cell nuclear antigen (PCNA) was investigated in PKH26^+^ and non-PKH26-retaining (PKH26^-^) cells in MKN45 and MKN74 spheres collected after 4 and 9 days of culture. Expression of both Ki67 and PCNA was significantly decreased in PKH26^+^cells (Figure 2B and 2C, Supplementary Figure 2A and 2B). These results indicated that PKH26^+^ CSC-like cells did not divide during sphere formation. Therefore, only these few PKH26-retaining CSC-like cells remained in a quiescent state in the spheres during their proliferation.

In spheres, 85.1±7.75% (n=3) and 81.9±9.11% (n=3) PKH26^+^ cells (red arrows) exhibited resistance to the combined drug treatment of 1 mM 5-fluorouracil (5-FU) and 10 μM paclitaxel (TAXOL) and radiation treatment (30 Gy) that are often used in clinical therapies respectively, whereas 97.42±0.64% (n=3) and 96.67±0.98% (n=3) PKH26^-^ cells were sensitive to these treatments and died, as determined by trypan blue staining (Figure 2D). These results indicated that CSC-like cells could maintain the quiescent state without any loss of viability for prolonged periods and resist chemoradiotherapy. Although a few PKH26^-^ cells (white arrows) survived with 2.57±0.64% (n=3) and 3.33±0.98% (n=3) after drug and radiation treatments respectively (Figure 2D), we speculated that these treatments could also induce some CSCs to enter the quiescent state and thereby become resistant to the treatments.

### Plk1 and Rsk1 are silenced in quiescent CSC-like cells and inhibition of Plk1 induces quiescence in CSC-like cells

**Figure 3:**
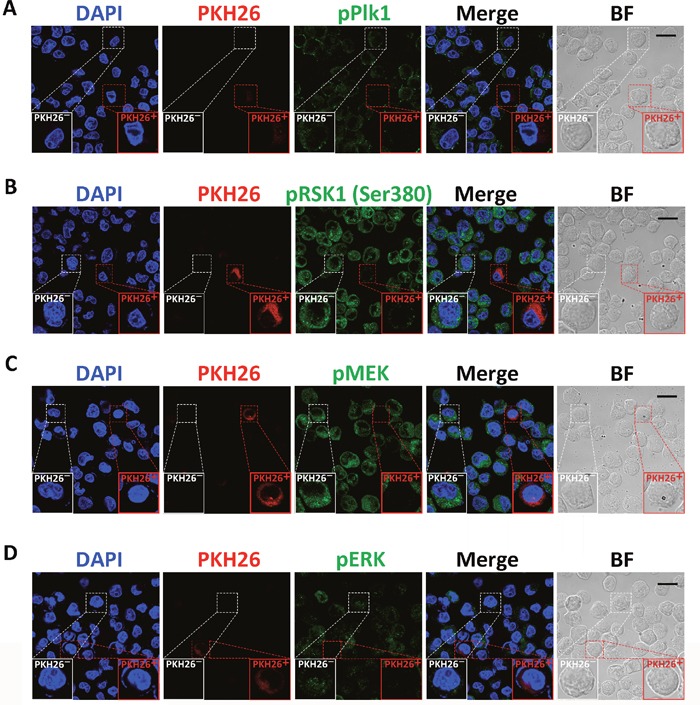
Analysis of the activities of Plk1, MEK, ERK, and RSK1 in quiescent CSC-like cells Immunofluorescence analysis of phosphorylation of Plk1 **(A)**, RSK1 **(B)**, MEK **(C)**, and ERK **(D)** in PKH26^+^ and PKH26^-^ CSC-like cells. Single CSC-like cells were labeled with PKH26 and cultured in serum-free medium for 7 days. DAPI stained the cell nuclei (blue). Red signals indicate PKH26 staining. Green signals show phosphorylation of the indicated proteins. BF = bright field. Scale bars, 20 μm. Experiments were repeated four times.

To study the regulation of CSC quiescence, we collected cells disaggregated from spheres originated from single PKH26-labeled MKN45 and MKN74 CSC-like cells and examined the phosphorylation level of Plk1 and RSK1 by immunofluorescence. The levels of phosphorylation of Plk1 and RSK1 decreased in PKH26^+^ cells compared with the PKH26^-^ cells (Figure 3A and 3B, Supplementary Figure 3). In addition, the activation of MEK and ERK was also inhibited in PKH26^+^ cells (Figure 3C and 3D, Supplementary Figure 3). These results indicated that the activities of Plk1 and MEK/ERK/RSK1 were suppressed in the quiescent CSC-like cells.

**Figure 4:**
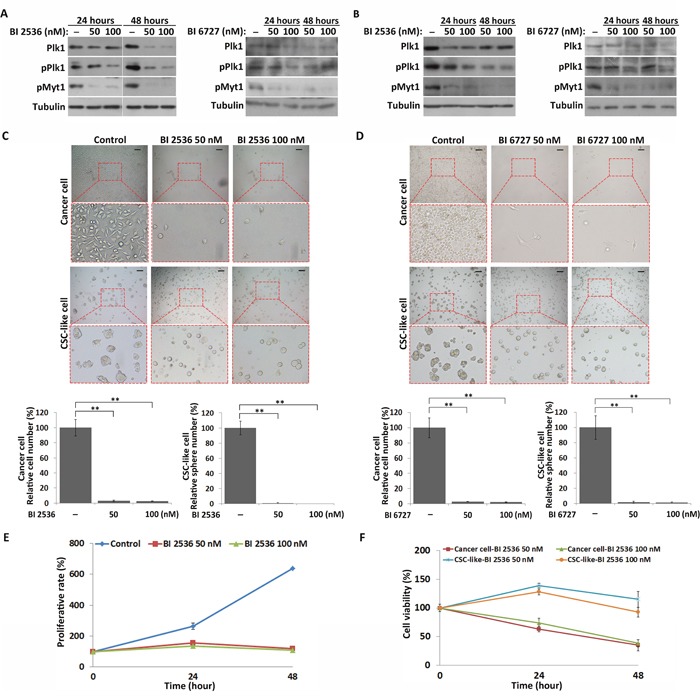
Inhibition of Plk1 induces quiescence in CSC-like cells, but apoptosis in cancer cells **(A)** and **(B)** Western blot analysis of the expression and phosphorylation of Plk1, and phosphorylation of Myt1 in the CSC-like and cancer cells after treatment with BI 2536 and BI 6727 for 24 and 48 hours, respectively. **(C)** and **(D)** Representative images of CSC-like and cancer cells treated with DMSO (control), BI 2536 and BI 6727 for 48 hours respectively. Informative regions are outlined by the red dotted box and enlarged (bottom panel). Scale bars, 50 μm. The number of cells and spheres were quantitatively analyzed from three independent experiments. All data represent the mean ± S.E. **, P < 0.05. **(E)** Proliferative rates of DMSO- and BI 2536-treated CSC-like cells as analyzed by CCK-8 assay. All data represent the mean ± S.E. (n=3). **(F)** Comparison of the BI 2536 sensitivities of CSC-like and cancer cells by CCK-8 assay. All data represent the mean ± S.E. (n=3).

To further examine the function of Plk1 in the regulation of CSC-like cell quiescence, Plk1 was inhibited by BI 2536 and BI 6727, two Plk1-specific inhibitors. CSC-like cells were disaggregated from spheres and cultured under non-adherent conditions in serum-free medium for 12 hours before treatment with these inhibitors. To confirm that treatment with BI 2536 and BI 6727 inhibits activation of Plk1 in MKN45 CSC-like cells and cancer cells, we measured the phosphorylation of Myelin transcription factor 1 (Myt1), a Plk1 substrate. Western blot analysis showed that Plk1 activity was successfully inhibited in MKN45 CSC-like and cancer cells after BI 2536 and BI 6727 treatment (Figure 4A and 4B). Compared with control cells, only 2.58±0.41% (n=3) and 2.28±0.38% (n=3) cells survived after 100 nM BI 2536 and BI 6727 treatment for 48 hours, respectively (Figure 4C and 4D). However, in the case of CSC-like cells, the spheres did not form, but cells were quiescent after BI 2536 and BI 6727 treatments (Figure 4C and 4D). Moreover, similar results were found in MKN74 cells (Supplementary Figure 4A and 4B). CSC-like cell proliferation was suppressed in the BI 2536-treated groups compared with the control group, according to the Cell Counting Kit-8 (CCK-8) assay (Figure 4E). These results indicated that inhibition of Plk1 induced quiescence in MKN45 and MKN74 CSC-like cells, but not in the cancer cells.

Next, we performed the CCK-8 assay to compare the BI 2536 sensitivities of MKN45 CSC-like and MKN45 cancer cells. Cancer cells were more sensitive to BI 2536 at two different concentrations than CSC-like cells. 33.4±6.34% (n=3) of cancer cells survived after BI 2536 treatment, while 92.63±8.24% (n=3) of CSC-like cells survived at the same time point following treatment of BI 2536 (Figure 4F). Moreover, BI 2536 and BI 6727-treated MKN45 and MKN74 cancer cells exhibited significantly more severe apoptosis than the CSC-like cells (Supplementary Figure 5). These results suggested that, in contrast with the cancer cells, the CSC-like cells enter the quiescent state in response to inhibition of Plk1.

### Involvement of Plk1 in CSC-like cell quiescence by inhibition of the MEK-ERK-RSK1 signaling pathway

**Figure 5:**
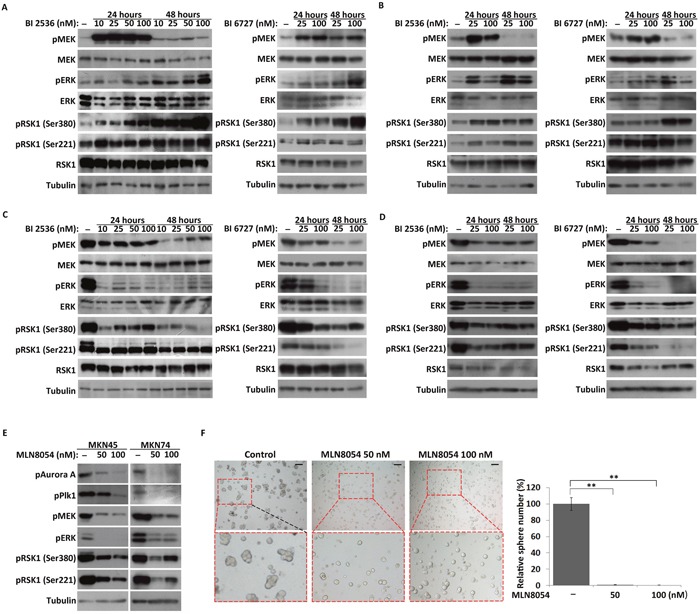
Effects of Plk1 inhibition on the MEK-ERK-RSK1 signaling pathway in CSC-like and cancer cells **(A)** and **(B)** Western blot analysis of the expression and activation of components of the MEK-ERK-RSK1 pathway in MKN45 and MKN74 cancer cells treated with BI 2536 and BI 6727 for 24 and 48 hours, respectively. **(C)** and **(D)** MKN45 and MKN74 CSC-like cells treated with BI 2536 and BI 6727 for 24 and 48 hours, respectively. **(E)** CSC-like cells treated with 50 and 100 nM MLN8054. α-Tubulin served as a loading control (bottom panel). **(F)** Representative images of MKN45 CSC-like cells treated with DMSO (control), 50 nM MLN8054 (Aurora A inhibitor), and 100 nM MLN8054. Scale bars, 50 μm. The number of spheres formed by MLN8054 treated cells and their control cells were quantified. All data represent the mean ± S.E (n=3). **, P < 0.05.

In our previous study, we found that Plk1 inhibits the MEK-ERK-RSK1 cascade during embryonic mitosis in *Artemia* species, and in HeLa cells [36]. Here, the effect of Plk1 inhibition on the MEK-ERK-RSK1 signaling pathway in both MKN45 and MKN74 CSC-like and cancer cells was examined. Consistent with our previous study in HeLa cells, phosphorylation of RSK1 and its upstream kinase MEK/ERK was significantly increased after Plk1 inhibition in cancer cells (Figure 5A and 5B). However, western blot analysis showed that the level of phosphorylation of RSK1 was significantly decreased after Plk1 inhibition in the CSC-like cells (Figure 5C and 5D). Meanwhile, the activation of MEK and ERK, upstream kinases of RSK1, was also suppressed in Plk1-inhibited CSC-like cells (Figure 5C and 5D). Based on these results, MKN45 and MKN74 CSC-like cells exhibited different regulation of RSK1 by Plk1 compared with the cancer cells.

As an upstream kinase of Plk1, Aurora A is implicated in important processes during cell proliferation [34, 35]. In this study, phosphorylation of Aurora A was inhibited by MLN8054, an Aurora A-specific inhibitor in CSC-like cells (Figure 5E). For CSC-like cells, spheres were not observed in the Aurora-inhibited groups in contrast with the control group (Figure 5F and Supplementary Figure 6). Similar to the results observed with Plk1 inhibitor treatment (Figure 5C and 5D), the MEK-ERK-RSK1 signaling pathway was inhibited in quiescent CSC-like cells upon treatment with MLN8054 (Figure 5E). These results indicated that Plk1 was involved in regulation of CSC-like cell quiescence via upstream Aurora A and the downstream MEK-ERK-RSK1 signaling pathway. Furthermore, the results confirmed that the regulation of RSK1 by Plk1 differed between MKN45/MKN74 CSC-like cells and cancer cells. Inhibition of Plk1 induced overall suppression of the MEK-ERK-RSK1 signaling pathway in the CSC-like cells, but over-activated ERK and RSK1 in the cancer cells.

### Inhibition of RSK1 activates Plk1 in CSC-like cells, but not in cancer cells

**Figure 6:**
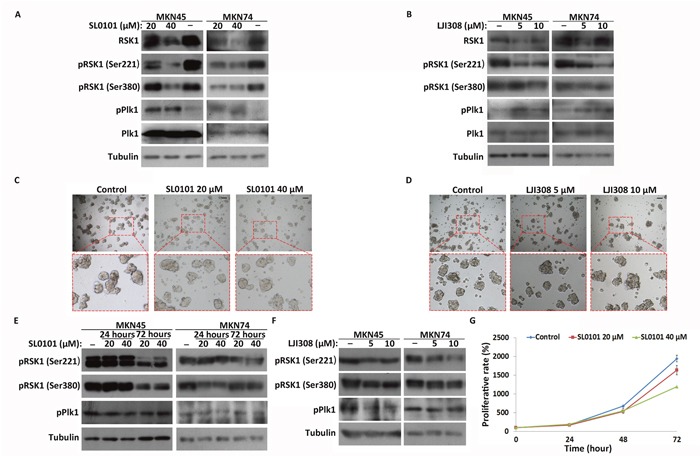
Inhibition of RSK1 in CSC-like and cancer cells and Plk1 response to inhibition **(A)** and **(B)** Western blot analysis of Plk1 and RSK1 activation in CSC-like cells after treatment with SL0101 and LJI308 for 72 hours. α-Tubulin served as a loading control (bottom panel). **(C)** and **(D)** Images of CSC-like cells treated with SL0101 (20 and 40 μM) and LJI308 (5 and 10 μM) for 4 days. The control group was treated with DMSO. Representative regions are outlined by red dotted boxes and enlarged (bottom panel). Scale bars, 50 μm. **(E)** and **(F)** Western blot analysis of RSK1 and Plk1 activation in cancer cells after treatment with SL0101 and LJI308. **(G)** Proliferative rates of MKN45 cancer cells treated with SL0101 and DMSO (control) were assessed using CCK-8 assay. All data represent the mean ± S.E (n=3).

To determine how RSK1 regulates CSC-like cell quiescence, activity of RSK1 was inhibited by SL0101 and LJI308, inhibitors of RSK1 (Figure 6A and 6B). The results showed that inhibition of RSK1 by the inhibitors had no effect on the formation of spheres by MKN45 and MKN74 CSC-like cells (Figure 6C and 6D, Supplementary Figure 7). Furthermore, western blot analysis showed that phosphorylation of Plk1 was significantly increased in the CSC-like cells after SL0101 and LJI308 treatments, in contrast with the control (Figure 6A and 6B). However, unlike the results observed in CSC-like cells, inhibition of RSK1 by SL0101/LJI308 had no effect on phosphorylation of Plk1 in MKN45 and MKN74 cancer cells (Figure 6E and 6F). Compared with control cells, the inhibitor reduced the proliferative rate of MKN45 cancer cells in a dose-dependent manner (Figure 6G). These results indicated that inhibition of RSK1 suppressed proliferation in the cancer cells, but not in the CSC-like cells. Plk1 activity did not change in response to inhibition of RSK1 in the cancer cells, but increased in the CSC-like cells.

**Figure 7:**
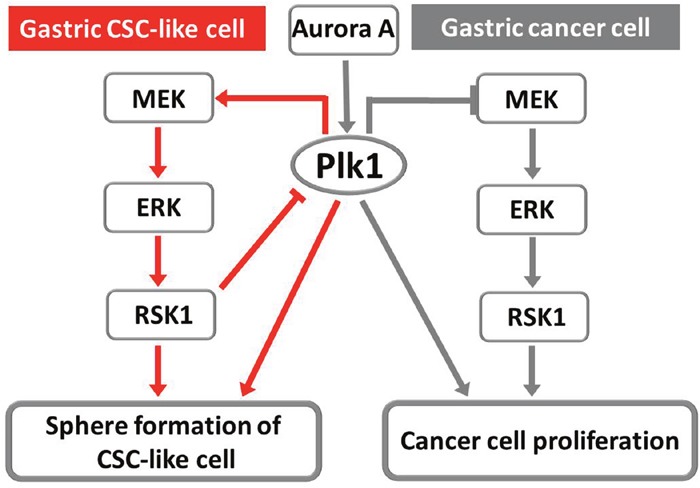
Two tentative models of the involvement of Plk1 and RSK1 in the regulation of sphere formation by gastric CSC-like cells and in the proliferation of cancer cells Red lines indicate molecular pathways in CSC-like cells, while gray lines indicate the molecular pathways in cancer cells and the common pathway.

Based on these results, we propose two tentative models for the involvement of Plk1 and RSK1 in regulating sphere formation and cellular proliferation in MKN45 and MKN74 cells (Figure 7). First, Aurora A activates Plk1 to enable successful sphere formation by the CSC-like cells and proliferation of the cancer cells. Inactivaition of the Plk1 pathway induces quiescence of CSC-like cells through suppression of RSK1 by its upstream signal-regulated kinases MEK and ERK. Conversely, inhibition of Plk1 in non-CSCs activates RSK1 through MEK/ERK and results in apoptosis and cell death. RSK1 activity, in turn, influences the activation level of Plk1 through a negative feedback mechanism in the CSC-like cells, but not in non-CSCs.

## DISCUSSION

In the present study, we found that both Plk1 and the MEK-ERK-RSK1 signaling pathway were silenced in quiescent CSC-like cells, indicating their involvement in the regulation of CSC quiescence. By treating MKN45 and MKN74 CSC-like cells with specific inhibitors of Plk1, Aurora A and RSK1, we found that Plk1 plays an important role in the activation of quiescent CSCs. Based on our results, these inhibitors could suppress proliferation and induce apoptosis in cancer cells. However, these inhibitors did not similarly affect quiescent CSC-like cells. Therefore, it is possible that quiescent CSCs could survive and lead to cancer relapse after treatments with these inhibitors and/or radiation.

Previous studies indicate that CSCs are more resistant to cancer therapies than differentiated and rapidly proliferating cancer cells. Breast CSCs were reported to display intrinsic resistance to chemotherapy by Li and coworkers [45]. Moreover, another group verified that the increased radioresistance of breast CSCs was due to a decreased level of reactive oxygen species in response to radiation [46]. Resistance of CSCs to therapy has also been found in several other cancer types, such as glioma, colorectal cancer, and chronic myeloid leukemia [47–49]. Here, we focused on the quiescence of CSCs, which is believed to be essential for CSCs to maintain their self-renewal capacity and resistance to chemotherapy. In contrast with MKN45 cancer cells, quiescent CSC-like cells in spheres were able to survive treatments with high concentrations of chemotherapeutic drugs and high-intensity radiation (Figure 2D, red arrows). Additionally, a few active CSC-like cells in spheres exhibited resistance to drugs and radiation, similar to the quiescent CSC-like cells (Figure 2D, white arrows). These results indicate that quiescent CSCs are resistant to drugs and radiation, and, more importantly, that some active CSCs could be induced to enter the quiescent state upon drug and radiation treatments.

In our previous study of *Artemia*, we found that Plk1 and RSK1 are both inactivated in embryonic cells released in the diapause phase (a state of obligate dormancy and non-division), but are highly active in nauplius larvae, in which cell division resumes [36]. Similar to quiescent CSCs, diapause embryos are characterized by the complete absence of cell division and DNA synthesis [50]. These results indicate that silencing of Plk1 and RSK1 plays important roles in inducing quiescence in both stem cells and CSCs. However, silencing of Plk1 and RSK1 by specific inhibitors induces apoptosis and death, in contrast with stem cells from *Artemia* diapause embryos and human CSC-like cells. These experiments imply that the regulation of Plk1 and RSK1 in stem cells is different from that in non-stem cells such as cancer cells. We propose that silencing of Plk1 and RSK1 induces stem cells to enter the quiescent state in response to severe environments such as drug and radiation treatments.

Evidence, including the results in this report, showed that SL0101 can inhibit the proliferation of cancer cells [40, 41]. However, more interestingly, inhibition of RSK1 by SL0101 and LJI308 had no effect on the proliferation and sphere formation of MKN45 and MKN74 CSC-like cells (Figure 6C and 6D, Supplementary Figure 7). Furthermore, western blot analysis showed that phosphorylation of Plk1 was significantly increased in the CSC-like cells after treatment with RSK1 inhibitors, in contrast with the cancer cells (Figure 6A and 6B). Plk1 and RSK1 formed a regulatory feedback mechanism in MKN45 and MKN74 CSC-like cells to help resist the loss of RSK1 functions, which differed from the mechanism in the cancer cells (Figure 7). Based on our results, CSCs can enter the quiescent state in response to unsuitable environments via the Plk1 and RSK1 signaling pathway, but cancer cells cannot. However, the molecular mechanisms underlying the differences in the regulation of the Plk1 and RSK1 signaling pathway between CSCs and cancer cells need to be elucidated.

In summary, inactivation of Plk1 induces quiescence of gastric CSC-like cells by inhibiting RSK1 activity. Importantly, regulation of Plk1 and RSK1 differed between gastric CSC-like cells and the cancer cells. Our findings may provide insights into the regulation of CSC quiescence, and show that Plk1 and RSK1 play important roles in the conversion of CSCs between active and quiescent states.

## MATERIALS AND METHODS

### Cell culture

The human gastric cancer cell lines MKN45 and MKN74 were purchased from Bioleaf Biotech. The cell lines were cultured in RPMI-1640 (Corning) supplemented with 10% fetal bovine serum (GIBCO) and antibiotics. CSC-like cells sorted from MKN45 and MKN74 cells were cultured in ultra-low attachment plates in Dulbecco's modified Eagle's medium: F12 (Corning) containing epidermal growth factor (EGF, 20 ng/ml), basic fibroblast growth factor (bFGF, 20 ng/ml), heparin sodium (5 ng/ml), and antibiotics. All cells were grown at 37°C in a humidified atmosphere with 5% CO_2_.

### FACS analysis of the surface marker CD44 in the gastric cancer cell lines

Cells were analyzed and sorted by FACS according to a previously described method [5, 7]. Cells were harvested using 0.25% trypsin and washed with phosphate-buffered saline (PBS). After centrifugation, the cells were suspended in PBS containing 10 μg/ml anti-CD44-FITC antibody (BD Biosciences) and 1% bovine serum albumin (BSA), and then incubated for about 1 hour at 37°C and gently pipetted every 15 minutes. Stained cells were washed twice, and resuspended in PBS containing 1 μg/ml propidium iodide, and then analyzed and sorted with a BD FACSAriaII flow cytometer.

### Spherical colony formation assay

FACS-sorted cells were plated in ultra-low attachment 6-well plates in the same medium described above at a density of 4000 live cells/well. The medium was replaced every 3 days. After 1-2 weeks, each well was examined using light microscopy and images of spherical colonies were taken.

### Immunofluorescence assay

Cells were fixed in 4% formaldehyde overnight and then washed with PBS. Fixed cells were permeabilized with 0.1% Triton X-100 prepared in PBS for 10 minutes. The cells were washed and blocked in antibody dilution buffer containing 1% BSA and 0.1% Tween 20/Triton X-100 for 30–60 minutes. Thereafter, the cells were incubated with the primary antibodies at a dilution of 1:100 overnight at 4°C. The slides were then washed twice with PBS and incubated with the secondary FITC-conjugated antibody (Invitrogen) in the dark for 2 hours at room temperature. Cell nuclei were stained with DAPI.

### PKH26 labeling assay

Cells were trypsinized, washed in PBS, and labeled with PKH26 (Sigma Aldrich) according to the manufacturer's instructions. In brief, 2×10^5^ cells were suspended in 200 μl of Diluent C and mixed with an equal volume of Diluent C containing 0.2 μl of PKH26 dye. The mixture was incubated for 5 minutes at room temperature, and then cells were washed with PBS three times. The labeled cells were cultured in serum-free medium in ultra-low attachment plates.

### Chemoresistance and radioresistance studies of quiescent Csc-like cells

CSC-like cells were disaggregated from spheres and labeled with PKH26 as described above. After about 10 days of culture in sphere-forming conditions, the resultant spheres were treated with 1mM 5-FU and 10 μM TAXOL (Sigma Aldrich) for 5 days. Viability was assessed by trypan blue staining.

For radioresistance experiments, spheres generated from PKH26-labeled CSC-like cells were irradiated with 30 Gy (1.2 Gy/min) using a Rad Source 2000 (Rad Source), and then the cells were cultured. The irradiated spheres were observed for 3-4 days, and viable cells were detected by trypan blue staining. Images of viable cells were taken by light and fluorescence microscopy (Nikon, ECLIPSE, TE2000-S).

### Inhibitor treatment

Trypsinized CSC-like cells and cancer cells of MKN45 and MKN74 were seeded in 6-well plates in the appropriate medium. After 12 hours, the cells were treated with the indicated concentrations of BI 2536 (Selleck), BI 6727 (Selleck), MLN8054 (Selleck), SL0101 (Tocris), and LJI308 (MedChem Express) and the corresponding medium was replaced every 24 hours. Images of cell growth were acquired by light microscopy. For western blot analysis, treated cells were harvested at the time points indicated below.

### Western blot analysis

Western blot analysis was performed as described previously [36]. Whole cell lysates were separated by 10% SDS-PAGE gels and then transferred to polyvinylidene fluoride membranes (Bio-Rad). After blocking in 1% blocking buffer for 1 hour, the membranes were incubated at 4°C overnight with specific primary antibodies, including anti-phospho-Plk1 (Abcam), anti-phospho (Ser380)-RSK1 and anti-phospho (Ser221)-RSK1(Cell Signaling Technology), anti-RSK (R&D Systems), anti-MAPK (p44/42 MAPK) and anti-phospho-MAPK (Cell Signaling Technology), and anti-phospho-MEK1/2 (Epitomics). An anti-tubulin antibody (Sigma Aldrich) was used as a loading control. Subsequently, appropriate secondary antibodies were applied. The immunoreactive bands were detected using a BM chemiluminescence western blotting kit (Roche).

### Cell proliferation assay

Cell proliferation and viability were assessed using the Cell Counting Kit-8 (CCK-8, Beyotime Biotechnology) as described in the manufacturer's instructions. Cells (2000 cells/well) were incubated in 96-well plates in triplicate for 12 hours before drug (BI 2536 and SL0101) treatments. Viable cells were quantified by measuring absorbance at a wavelength of 450 nM with a Multiskan EX plate reader (Thermo Fisher Scientific).

### Apoptosis detection

Apoptotic cells were detected using the TdT-mediated dUTP nick end labeling (TUNEL) assay following the manufacturer's instructions (DeadEnd™ Colorimetric TUNEL System, Promega). Cells were treated with 50 nM BI 2536 and BI 6727 for 24 hours and fixed in 4% formaldehyde for TUNEL detection. Images of TUNEL-stained cells were recorded using light microscopy. The proportion of TUNEL-stained cells was calculated by counting cells in three individual views randomly.

## SUPPLEMENTARY MATERIALS FIGURES


