# Supplementary material for: Involvement of Polo-like kinase 1 (Plk1) in quiescence regulation of cancer stem-like cells of the gastric cancer cell lines
Source: Oncotarget. 2017 Apr 5;8(23):37633–45. doi: 10.18632/oncotarget.16839 (PMC5514936; doi:10.18632/oncotarget.16839)
Supplement: Supplementary file 1 [file oncotarget-08-37633-s001.pdf]

Involvement of Polo-like kinase 1 (Plk1) in quiescence regulation of cancer stem-like cells of the gastric cancer cell lines

SUPPLEMENTARY MATERIALS

SUPPLEMENTARY FIGURES

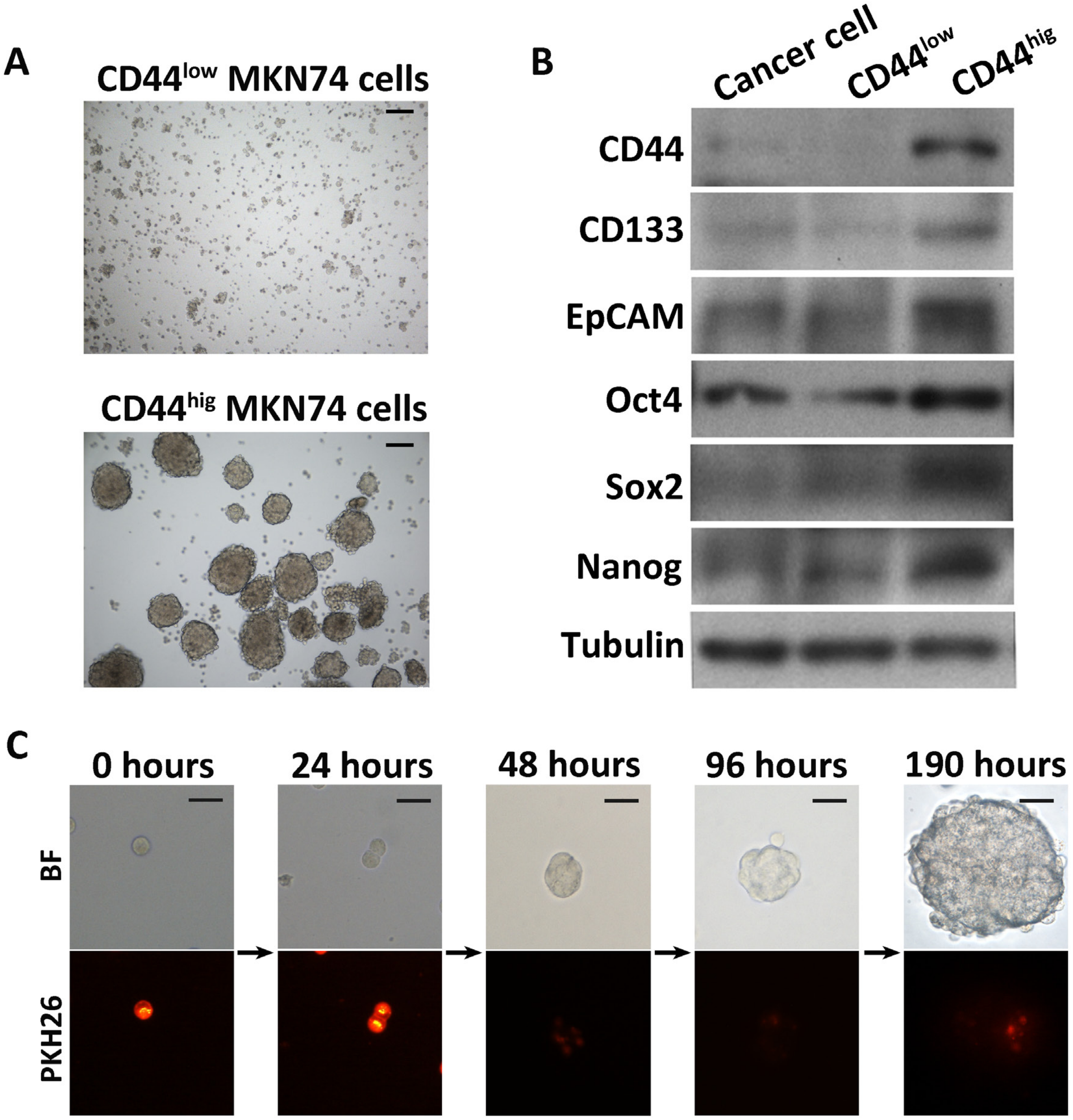

Supplementary Figure 1: Isolation and characterization of CSC-like cells from MKN74 cells. **(A)** Representative images of CD44^{low} and CD44^{hig} MKN74 cells culturedin serum-free medium for 10 days. Scale bars, 50 µm. **(B)** Expression profiles of stem cell markers in CD44^{hig}, CD44^{low}, and unsorted cells detected by Western blot. **(C)** The PKH26-labeling retention assay was performed upon MKN74 CSC-like cell sphere formation. Single CSC-like cells were labeled with PKH26 and cultured in serum-free medium for 8 days. Images were taken at the indicated time points. Red signals indicate PKH26 staining. BF = bright field. Scale bars, 25 µm.

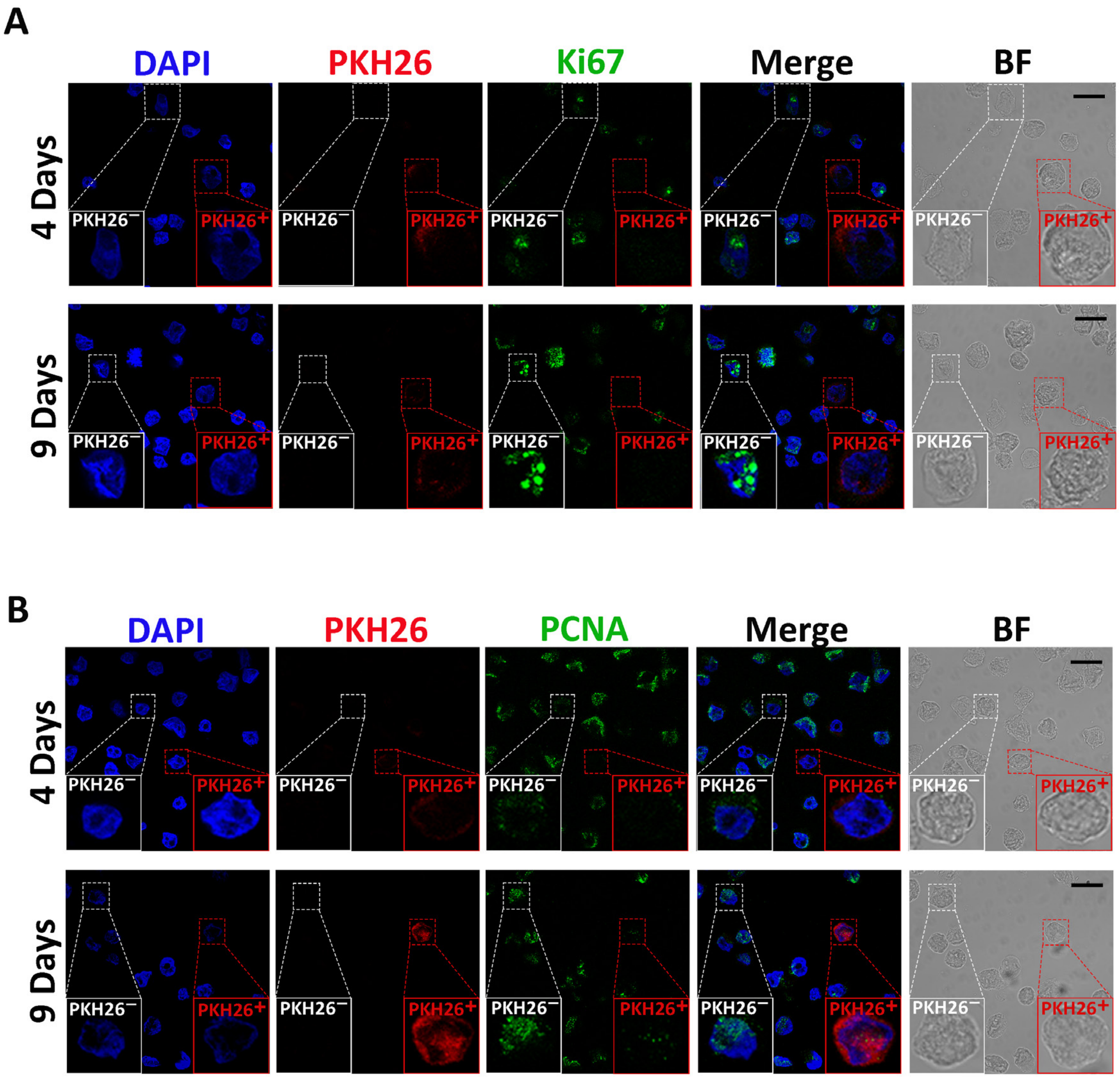

Supplementary Figure 2: Identification of quiescent CSC-like cells in MKN74 spheres. Expression of Ki67 **(A)** and PCNA **(B)** in PKH26^{+} and PKH26^{-} MKN74 CSC-like cells. DAPI stained the cell nuclei (blue). Red signals indicate PKH26 staining. Green signals show expression of Ki67 and PCNA. BF = bright field. Scale bars, 20 µm.

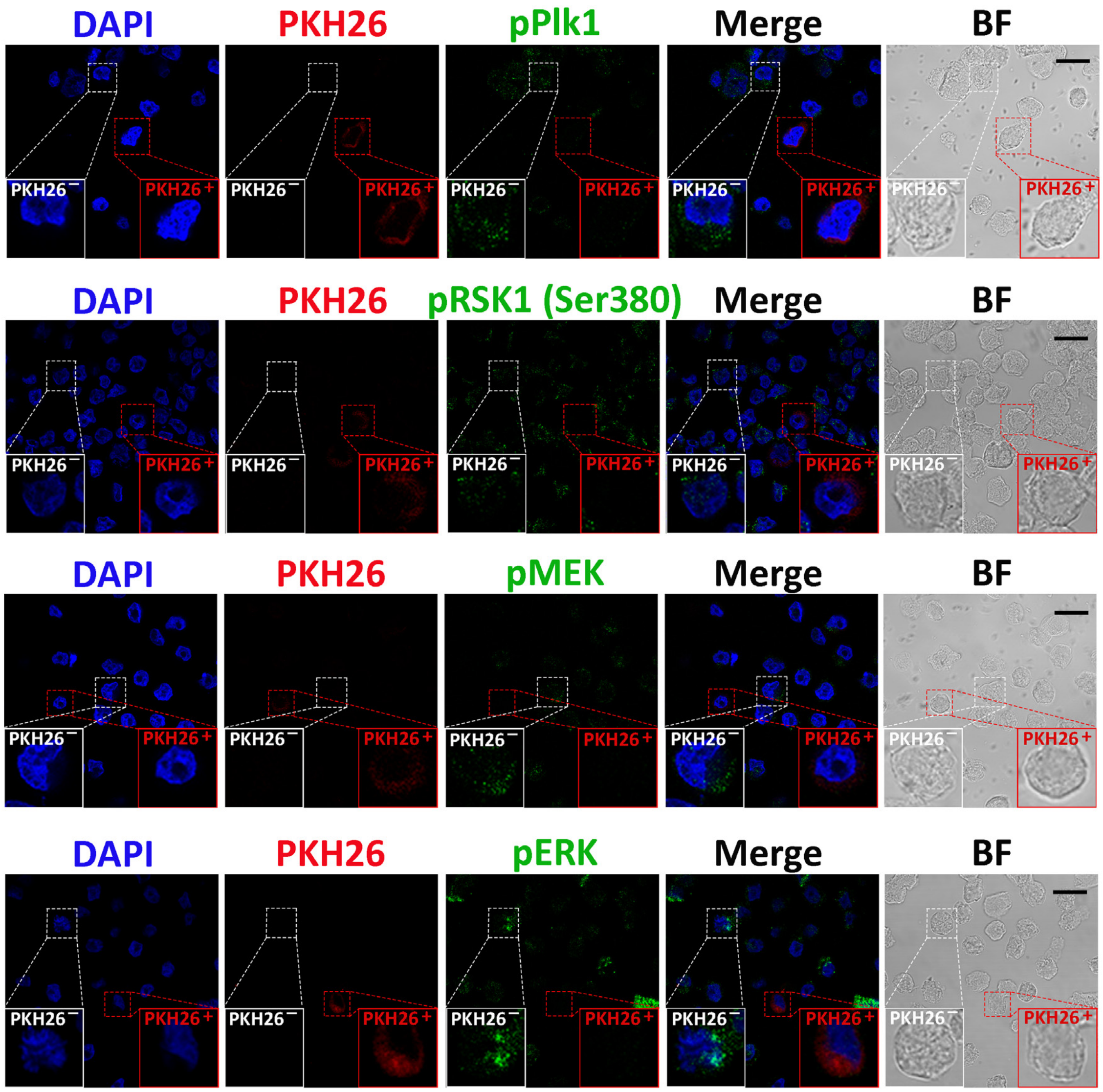

Supplementary Figure 3: Analysis of the activities of Plk1, RSK1, MEK, and ERK in quiescent MKN74 CSC-like cells. Immunofluorescence analysis of phosphorylation of Plk1,RSK1,MEK, and ERK in $PKH26^{+}$ and $PKH26^{-}$ MKN74 CSC-like cells. DAPI stained the cell nuclei (blue). Red signals indicate PKH26 staining. Green signals show phosphorylation of the indicated proteins. BF = bright field. Scale bars, 20 μm.

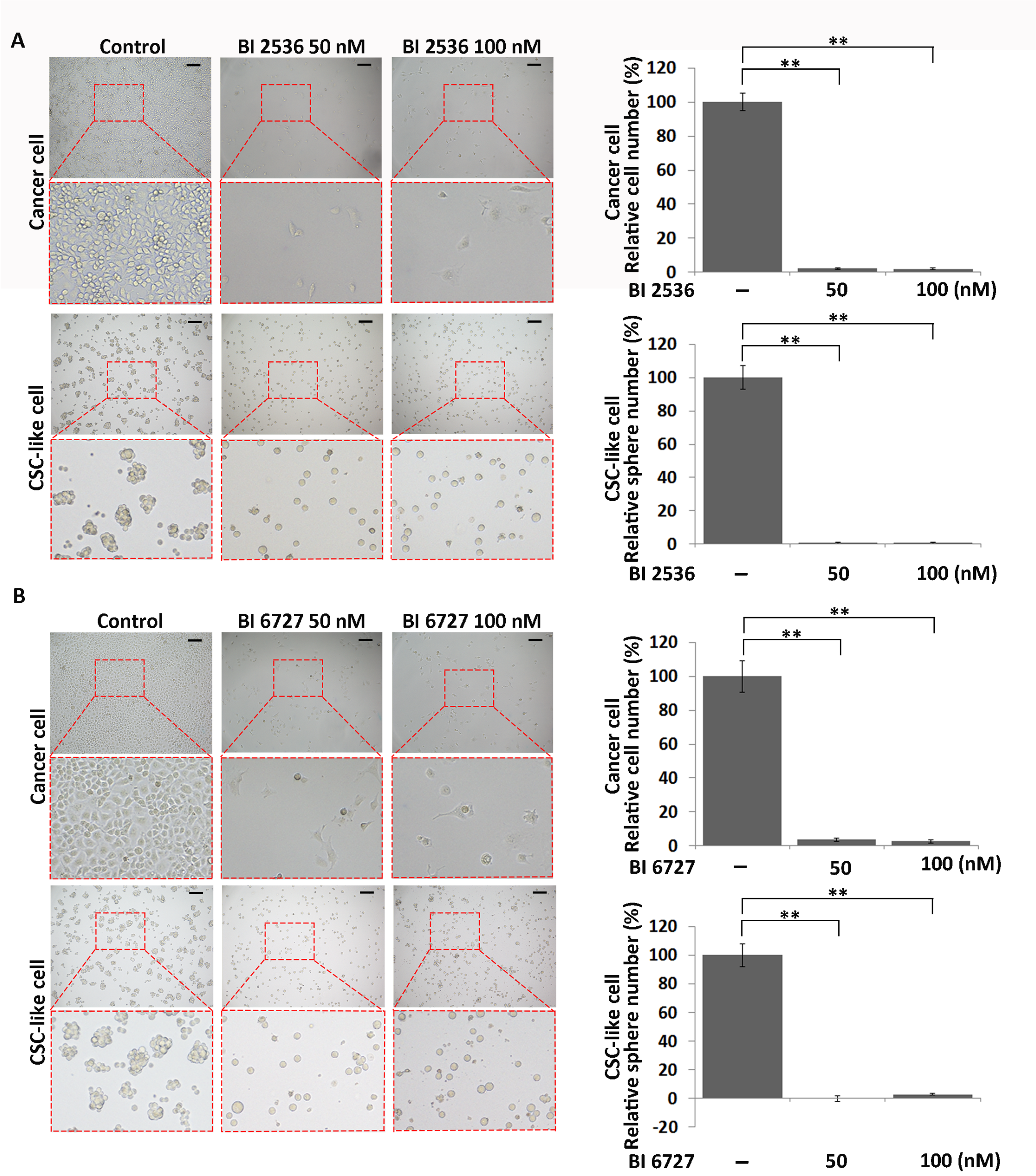

Supplementary Figure 4: Inhibition of Plk1 suppresses proliferation of MKN74 cancer cells and sphere formation of MKN74 CSC-like cells. **(A)** and **(B)** Representative images of BI 2536- and BI 6727-treated MKN74 cancer cells and CSC-like cells after 48 hours. Scale bars, 50 μm. The numbers of cells and spheres were quantified. Data are the means ± S.E. of three independent experiments in A to B. **, $P < 0.05$.

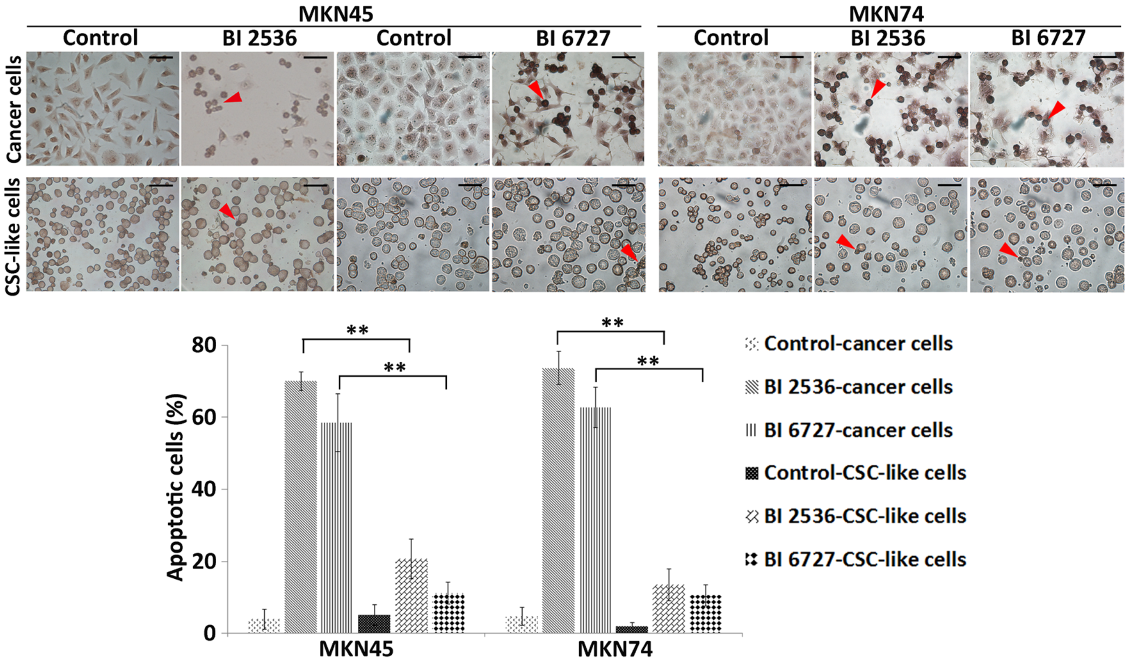

Supplementary Figure 5: Apoptotic analysis of BI 2536- and BI 6727-treated MKN45 and MKN74 CSC-like and cancer cells by TUNEL assay. TUNEL-stained cells are indicated by red arrows. Scale bars, 50 µm.The relative numbers of apoptotic cells among BI 2536- and BI 6727-treated CSC-like and cancer cells were quantified (bottom panel). All data represent the mean ± S.E (n=3). **, $P < 0.05$.

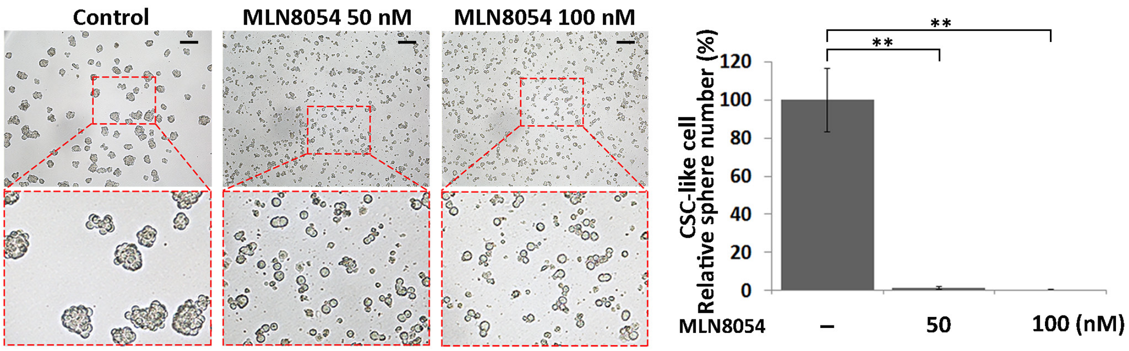

Supplementary Figure 6: Inhibition of Aurora A suppresses sphere formation in MKN74 CSC-like cells. Representative images of MKN74 CSC-like cells treated with DMSO (control), 50 nM MLN8054 (Aurora A inhibitor), and 100 nM MLN8054. Scale bars, 50 µm. The numbers of spheres formed by MLN8054 treated cells and control cells were quantified. All data represent the mean ± S.E (n=3). **, $P < 0.05$.

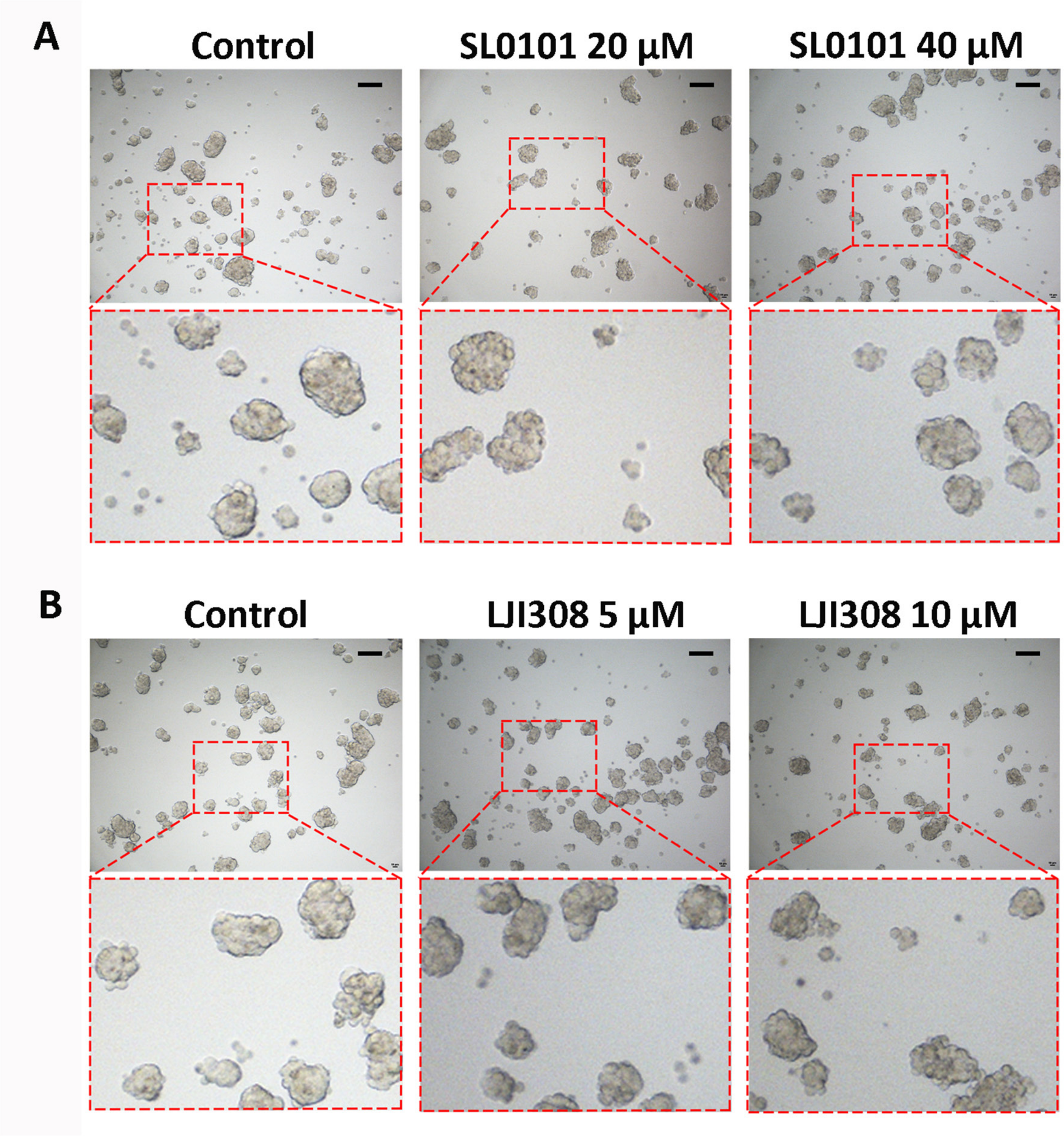

Supplementary Figure 7: Effects of RSK1 inhibition on sphere formation in MKN74 CSC-like cells. **(A)** and **(B)** Representative images of MKN74 CSC-like cells treated with SL0101 (20 and 40 μM) and LJI308 (5 and 10 μM) for 4 days. The control group was treated with DMSO. Scale bars, 50 μm.
